# The Microbiota–Diet–Immunity Axis in Cancer Care: From Prevention to Treatment Modulation and Survivorship

**DOI:** 10.3390/nu17172898

**Published:** 2025-09-08

**Authors:** Sabrina Tini, Jessica Baima, Stella Pigni, Valentina Antoniotti, Marina Caputo, Elena De Palma, Luigi Cerbone, Federica Grosso, Marta La Vecchia, Elisa Bona, Flavia Prodam

**Affiliations:** 1 Department of Health Science, University of Piemonte Orientale, 28100 Novara, Italy; sabrina.tini@uniupo.it (S.T.); jessica.baima@uniupo.it (J.B.); marina.caputo@uniupo.it (M.C.); marta.lavecchia@uniupo.it (M.L.V.); 2 Department of Translational Medicine, University of Piemonte Orientale, 28100 Novara, Italy; 20007328@studenti.uniupo.it (S.P.); valentina.antoniotti@uniupo.it (V.A.); 20032250@studenti.uniupo.it (E.D.P.); 3 Unit of Endocrinology, University of Piemonte Orientale, 28100 Novara, Italy; 4 Mesothelioma, Melanoma and Rare Cancer Unit, Azienda Ospedaliera Universitaria SS Antonio e Biagio e Cesare Arrigo, 15121 Alessandria, Italy; luigi.cerbone@ospedale.al.it (L.C.); federica.grosso@ospedale.al.it (F.G.); 5 Department for Sustainable Development and Ecological Transition, Università del Piemonte Orientale, 13100 Vercelli, Italy; elisa.bona@uniupo.it; 6 Center for Translational Research on Autoimmune and Allergic Diseases, University of Piemonte Orientale, 28100 Novara, Italy; 7 Simple Departmental Structure Research Laboratories-Integrated Activities Research and Innovation Department, Azienda Ospedaliera SS. Antonio e Biagio e Cesare Arrigo, 15121 Alessandria, Italy

**Keywords:** cancer, microbiota, immunotherapy, fecal transplant, diet, survivors

## Abstract

Growing evidence highlights the pivotal role of the gut microbiota in cancer development, progression, response to therapy, and survivorship. Diet plays a central role in shaping gut microbiota composition, influencing the immune system and overall host health. Plant-based diets and the Mediterranean diet promote health-associated microbial communities that increase the production of several metabolic-end products, including short-chain fatty acids that support mucosal barrier integrity, anti-inflammatory effects, and modulation of the immunity of the host. Conversely, Western dietary patterns promote cancer progression and negatively impact the response to standard treatments. Furthermore, gut microbiota influences the effectiveness of cancer therapies, including chemotherapy, radiotherapy and, mainly, immunotherapy. Modulating microbial species, their metabolites, or their activities in the cancer microenvironment through dietary interventions, common or engineered probiotics, prebiotics, postbiotics, antibiotics or fecal microbial transplant are emerging as promising strategies for cancer prevention and tailored management in survivorship. In this review, we explore the intricate interplay between diet, gut microbiota, and cancer, focusing on how specific microbial communities’ impact therapeutic outcomes, and the challenges in the modulation of the microbiota environment through several interventions, including diet. This emerging paradigm paves the way for integrating nutrition and microbiota-targeted strategies as innovative tools in the context of precision medicine.

## 1. Introduction

In recent years, a growing body of research has highlighted the significant impact of the gut microbiota and microbiome on human health [1,2]. Although often used interchangeably, the terms describe distinct concepts. The microbiota refers to the diverse community of microorganisms, including bacteria, fungi, viruses, and protozoa, that inhabit various body niches [1]. Among these, the gut microbiota is the most abundant and complex, often regarded as a “virtual organ” due to its extensive metabolic and immunological functions [1,3]. In contrast, the microbiome encompasses the collective genetic material of these microorganisms, including their structural elements, metabolic products, and functional interactions with the host [1,4].

The gut microbiota plays a crucial role in regulating host metabolism, supporting immune system function, and defending against pathogenic organisms [1]. Its development begins at birth and is shaped by multiple factors, including gestational age, delivery mode (vaginal or cesarean), feeding practices (breastfeeding vs. formula), and early-life antibiotic exposure [5]. During the first weeks of life, the microbiota is primarily dominated by anaerobes such as *Bifidobacterium*, *Bacteroides*, *Clostridia*, and *Parabacteroides* [5]. By age two, its composition stabilizes and contributes significantly to immune maturation. By infancy, it starts to resemble the adult microbiota, dominated by six major phyla, particularly *Bacillota* (*synonym Firmicutes*) and *Bacteroidota* (*synonym Bacteroidetes*) [1,5,6]. However, the gut microbiota composition continues to evolve with age, showing some differences in microbial composition during pre-adolescence and adolescence. For instance, pre-adolescents exhibit species involved in the synthesis of vitamin B12 and de novo folate synthesis. Moreover, further shifts in microbiota composition occur during aging [5].

Defining a “healthy” microbiota remains challenging due to high inter-individual variability [1]. However, high microbial diversity is widely recognized as a marker of a resilient and balanced gut ecosystem. Disruption of this balance, termed “dysbiosis”, has been linked to increased susceptibility to metabolic disorders, inflammatory diseases, and cancer [1,7].

Extending this understanding, recent studies have emphasized the pivotal role of gut microbiota in carcinogenesis. Alongside genetic predisposition, environmental pollutants, and dietary habits, alterations in gut microbial communities are now considered among the emerging hallmarks of cancer [2,7]. Microbes in the gut interact with host immune pathways, particularly through Toll-like receptor 4 (TLR4) on intestinal epithelial and immune cells, contributing to both local and systemic inflammation. This dysregulated immune signaling can disrupt the delicate cross-talk between immune and stromal cells, creating a microenvironment suitable for tumor initiation and progression [6,8]. Furthermore, dysbiosis favors the overgrowth of pro-inflammatory bacteria that release metabolites and toxins, which exacerbate mucosal inflammation and promote tumor development [2,8].

Among external modulators of gut microbiota, diet is without doubt the most influential [9]. The Western diet (WD), characterized by high consumption of refined sugars, saturated fats, ultra-processed foods, and low intake of fiber, fruits, and vegetables, is consistently associated with microbial imbalance, low-grade systemic inflammation, and increased cancer risk. This dietary pattern supports the expansion of pathobionts (i.e., *Prevotella* and *Desulfovibrio*) and the reduction in beneficial commensals, to a pro-tumorigenic environment [9,10]. One of the peculiar characteristics of the WD is its deficiency in microbiota-accessible carbohydrates (MACs), which play a key role in sustaining gut microbial diversity and metabolic function [11]. The lack of MACs compromises the production of beneficial microbial metabolites and predisposes the host to immune dysregulation and chronic inflammatory conditions [6,11]. Similarly, an animal-based diet rich in red meat has been shown to promote systemic inflammation through the production of diet-derived microbial metabolites. Among these, there are deoxycholic acid (DCA), trimethylamine *N*-oxide (TMAO), and branched-chain amino acids (BCAAs), all of which have been implicated in adverse health outcomes [12], including insulin resistance, metabolic syndrome, and cardiometabolic diseases [6,13,14,15], as well as in tumor initiation and progression [16,17,18,19].

Conversely, fiber-rich diets, such as the Mediterranean diet (MD) and particularly plant-based diets (PBD), are associated with increased microbial diversity and the enrichment of health-promoting species such as *Bifidobacterium* and *Lactobacillus* [9,20,21]. These microbes ferment dietary fibers like inulin, producing short-chain fatty acids (SCFA), butyrate, acetate, and propionate, which exert a range of health benefits. Butyrate, in particular, has anti-inflammatory, immunomodulatory, and anti-carcinogenic properties. It promotes the development of regulatory T cells contributing to the inhibition of cancer cells proliferation. Thus, diets rich in fiber can promote a gut microbiome that supports immune regulation and contributes to protection against carcinogenesis [6,20].

Notably, the gut microbiota not only influences tumor initiation, but also modulates the response to cancer therapies, including immune-checkpoint inhibitors (ICIs) [12,16]. These findings underscore the potential of gut microbiota composition as both a predictive and prognostic biomarker, aiding in the stratification of patients for personalized cancer treatment [2,6].

Furthermore, growing evidence supports the role of the gut microbiota as a key mediator between diet and several outcomes relevant to cancer survivorship care, including treatment-related toxicities, comorbidities, and overall quality of life [22,23,24,25].

This review aims to provide a detailed examination of the complex interplay between diet, gut microbiota, and the immune system across the entire cancer care continuum, from prevention to treatment and survivorship. Specifically, by integrating current evidence from preclinical and clinical studies, we aim to highlight the potential role of diet-mediated microbiota modulation as a strategy to improve cancer prevention, treatment efficacy, and long-term survivorship care.

## 2. Anti- and Pro-Tumorigenic Effects of the Microbiota

As mentioned above, the complex and dynamic microbial ecosystem in the human gastrointestinal tract is key in maintaining host homeostasis and contributes significantly to the pathogenesis of various diseases, including cancer [26]. Indeed, the gut microbiota is emerging as a key modulator in cancer biology, exerting both anti-tumorigenic and pro-tumorigenic effects [27]. These opposing roles are associated with distinct immunological and inflammatory pathways [28]. The anti-tumorigenic effects of the gut microbiota can be classified into three main mechanisms: competition with pro-tumorigenic species for ecological niches, the production of beneficial metabolites, and the enhancement of anti-tumor immune responses. Conversely, the pro-tumorigenic effects involve the induction of genotoxicity via microbial metabolites or toxins, and the promotion of chronic inflammation that contributes to a tumor-supportive environment [29].

### 2.1. Anti-Tumorigenic Effects of Microbiota

#### 2.1.1. Competition with Pro-Tumorigenic Species

Commensal bacteria have developed adaptive mechanisms to thrive in the gut environment, largely by efficiently utilizing available nutrients. Beneficial species can compete with pro-tumorigenic bacteria for both ecological niches and nutrient resources, creating an unfavorable environment. Probiotics such as *Lactobacillus acidophilus*, *Lactobacillus rhamnosus*, and *Lactobacillus fermentum* have been shown to alleviate clinical symptoms of inflammatory bowel disease (IBD) and may also attenuate early-stage cancer development [30,31]. Furthermore, Chou et al. demonstrated that dietary supplementation with *Lactobacillus* can help restore balance in the gut microbial community by suppressing *Bacteroidetes*, contributing to the inhibition of colon tumorigenesis in mice [32].

In colorectal cancer (CRC), changes in the intestinal microbiota composition have been observed, suggesting a close correlation between dysbiosis and tumor initiation and progression. Thus, beneficial bacterial metabolites can activate signaling pathways involved in cell survival [33]. Ferrichrome, a siderophore secreted by *Lactobacillus casei*, was shown to induce apoptosis in CRC cells through a JNK-dependent mechanism involving the activation of DNA damage-inducible transcript 3 (DDIT3) [34].

Modulating specific microbial metabolic pathways or nutrient requirements has become a promising approach to selectively suppress pathogenic bacteria associated with cancer progression. Tungstate has been identified as a specific inhibitor of the molybdenum cofactor-dependent microbial respiratory pathways. This metabolic pathway, selectively utilized by pathogenic *Enterobacteriaceae* such as *E. coli* under inflammatory conditions, represents a therapeutic target whose inhibition has been shown to mitigate colitis and *E. coli*-associated colorectal tumorigenesis [35].

#### 2.1.2. Anti-Tumor Immune Responses

Gut microbiota is fundamental in orchestrating immune system development, modulating the activation thresholds and functional responsiveness of both innate and adaptive immune cells. Specific taxa, such as *Bacteroides fragilis* (NTBF) strain, *Akkermansia muciniphila*, *Clostridium* spp., and *Lactobacillus rhamnosus*, have been shown to stimulate dendritic cell maturation and enhance antigen presentation, thereby promoting the activation and clonal expansion of CD8^+^ cytotoxic T lymphocytes and T helper 1 (Th1)-polarized CD4^+^ T cells [36,37,38]. These beneficial bacteria can infiltrate the tumor microenvironment, supporting immune surveillance and mediating cytolytic activity against neoplastic cells. In addition to promoting effector responses, *Lactobacillus reuteri*, *Bacteroides fragilis* and *Clostridium* spp. have been found to regulate the production of FoxP3^+^ regulatory T cells (Treg), which are essential for limiting inflammatory pathways and may attenuate tumor progression [39,40,41].

Microbial signals can potentiate the secretion of cytokines with immunostimulatory and antitumor functions, such as IL-12, IL-22, TNF, and IFN-γ, potentially through the activation of innate immune pathways mediated by pathogen-associated molecular patterns (PAMPs) [42]. Among these, IL-22 has emerged as a key player in maintaining mucosal barrier integrity, particularly in the skin, gastrointestinal tract, and respiratory tract [43]. In the context of cancer, IL-22 plays a dual role; while it could be beneficial in supporting mucosal defense, excessive IL-22 signaling has been implicated in colorectal carcinogenesis. In murine models of CRC, elevated IL-22 levels correlate with increased tumor burden and reduced survival, potentially through the promotion of cellular proliferation and inflammation [44,45]. *Bacteroides dorei*, *Parabacteroides distasonis*, and *Paraprevotella xylaniphila* have been shown to promote IFN-γ production by CD8^+^ T cells, further enhancing antitumor immunity in murine models. Additionally, *Bifidobacterium bifidum* can restore immune balance by enhancing regulatory pathways [46]. Lastly, some microbial antigens may mimic tumor neoantigens, leading to T cell cross-reactivity through molecular mimicry and reinforcing tumor-specific immune responses [42,43].

#### 2.1.3. Beneficial Metabolites

Homeostatic interactions between the microbiota and the immune system are largely mediated by bacterial metabolites, particularly SCFA, such as butyrate and propionate, produced by commensal bacteria during the fermentation of dietary fiber [47]. SCFA exert immunomodulatory effects through G protein–coupled receptors and histone deacetylase inhibition, influencing both local and systemic immune responses. They enhance cytotoxic T lymphocyte activity, support the recruitment and functional activation of antigen-presenting cells, and promote a pro-inflammatory macrophage phenotype conducive to tumor immunosurveillance [48]. Additionally, SCFA stimulate the production of key cytokines such as IL-6 and IL-12 and facilitate the activation of dendritic cells and neutrophils, thereby contributing to the establishment of an immunostimulatory tumor microenvironment and reinforcing anti-tumor immune mechanisms [49].

For instance, butyrate enhances Treg development, stabilizes the intestinal epithelial barrier, reducing gut permeability and consequently lowering the risk of metastatic dissemination [50]. SCFA promotes the expansion of Treg and supports antimicrobial functions of intestinal macrophages, thereby sustaining immune homeostasis and counteracting tumor-promoting inflammation. SCFA can also act directly on cancer cells; for instance, *Faecalibaculum rodentium* and its human counterpart *Holdemanella biformis* produce SCFA that inhibit calcineurin–NFATc3 signaling, modulate protein acetylation, and suppress tumor cell proliferation [51].

Beyond SCFA, other microbial-derived metabolites influence anti-tumor immunity and therapeutic efficacy. Indole derivatives from tryptophan metabolism, secondary bile acids, and 3-indolepropionic acid (IPA) have been implicated in modulating mucosal immunity and cytokine profiles [52,53]. Additionally, *Lactobacillus reuteri* and its metabolite reuterin exert antiproliferative effects on CRC cells by inducing protein oxidation and impairing ribosomal biogenesis and translation [54].

These insights underscore the multifaceted role of microbiota-derived metabolites in reinforcing mucosal immunity, preserving epithelial barrier function, and enhancing antitumor responses, representing potential targets for innovative strategies in cancer prevention and immunotherapy.

### 2.2. Pro-Tumorigenic Effects of Microbiota

#### 2.2.1. Genotoxicity

Accumulating evidence highlights the role of specific gut microbes in initiating cancer through direct genotoxic mechanisms [29]. For instance, *Escherichia coli* produces colibactin, a polyketide–peptide genotoxin capable of inducing DNA double-strand breaks and activating DNA damage responses in host cells [55]. In interleukin (IL)-10-deficient mouse models, colonization with *E. coli* strains carrying the polyketide synthase (PKS) genomic island significantly increases tumor incidence, while strains lacking this cluster fail to elicit the same effect, underscoring the inflammation-independent contribution of microbial genotoxins to carcinogenesis. The PKS gene cluster is more frequently detected in the colonic mucosa of CRC patients compared to healthy individuals [55,56]. Similarly, *Bacteroides fragilis* strains expressing *B. fragilis* toxin (BFT) have been shown to facilitate tumorigenesis in Apc (min/+) mice through IL-17-mediated inflammatory pathways that depend on STAT3 activation [57]. Other toxins, such as cytolethal distending toxin (CDT) and cytotoxic necrotizing factor 1 (CNF1), also contribute to genotoxic stress by disrupting cell cycle progression and promoting chromosomal instability [58].

Gut microbiota not only produces genotoxins (as colibactin, CNF1), but also induces chronic inflammation, which increases the production of reactive oxygen species (ROS) and reactive nitrogen intermediates (RNI) by immune cells [29,59]. These reactive molecules contribute to oxidative DNA damage and genomic instability, supporting a tumor-promoting microenvironment.

#### 2.2.2. Inflammation

Chronic inflammation plays a central role in tumor initiation and progression, particularly within the gastrointestinal tract [60,61]. Dysbiosis can compromise mucosal barrier integrity and increase epithelial permeability, facilitating the translocation of microbial products into host tissues. This process activates pattern recognition receptors (PRRs), including Toll-like receptors (TLRs), on immune and epithelial cells, triggering the release of pro-inflammatory cytokines such as IL-6, IL-12, IL-17, IL-22, IL-23, TNF-α, and IL-1β [62,63]. These cytokines stimulate oncogenic signaling pathways (NF-κB, STAT3, Wnt, and Notch), promoting epithelial proliferation, immune evasion, and malignant transformation [64]. Notably, IL-22-producing CD4^+^ T cells have been shown to enhance colorectal cancer cell stemness, with colonic dendritic cells contributing to local IL-22 production independent of their maturation state [65,66].

In models of colitis-associated cancer, persistent inflammation recruits and activates innate immune cells, including neutrophils, macrophages, and dendritic cells, which release ROS that exacerbate DNA damage and accelerate cellular turnover [60,67]. Additionally, TLR4 overexpression induces COX-2, a well-established marker of inflammation-associated colorectal cancer and inflammatory bowel disease [68]. Gut microbiota also amplifies systemic inflammation by modulating acute phase proteins such as serum amyloid A (SAA) and *C*-reactive protein (CPR) [69,70,71]. This phenomenon is particularly pronounced in the presence of segmented filamentous bacteria like *Candida albicans* and *Citrobacter rodentium*, which promote pathogen clearance by inducing T helper 17 (Th17) cell responses in the lamina propria and recruiting neutrophils and other immune cells, thereby enhancing mucosal immune activation [41,72].

Beyond inflammation, dysbiosis also contributes to immune dysregulation that favors tumor progression. Polyamines inhibit lymphocyte proliferation and facilitate tumor invasion via mechanisms resembling tumor-derived proteases [73]. Imidazole propionate, often elevated in dysbiosis, activates the mTOR signaling pathway, which is implicated in oncogenesis and immune evasion [74]. Systemic immune imbalance is further evidenced by altered neutrophil-to-lymphocyte ratios, which correlate with poor outcomes in cancers such as early-stage breast cancer [75]. Moreover, cytotoxic CD8^+^ T cells frequently exhibit functional impairment in dysbiotic states, reducing effective tumor control. Sustained stimulation of TLRs by microbial antigens may instead promote tumor progression by perpetuating chronic inflammation, cytokine secretion, and extracellular matrix remodeling [60].

Importantly, microbial-driven inflammation and immune dysregulation are not limited to CRC. Intestinal dysbiosis, characterized by increased abundance of pro-inflammatory and genotoxic species, such as *Staphylococcus* and *Escherichia coli*, along with reduced microbial diversity, has also been observed in breast cancer patients [76]. Such alterations are associated with chromosomal instability, immune dysfunction, and delayed diagnosis, suggesting that microbiota-induced inflammation may contribute to tumorigenesis even beyond the gastrointestinal tract.

## 3. Diet–Microbiota Interaction in Tumorigenesis

The intricate and dynamic relationship between diet, gut microbiota, and cancer has garnered increasing attention, establishing a critical axis for disease prevention and progression [77]. Diet, as a modifiable environmental factor, profoundly shapes the composition and function of the gut microbiota, which in turn influences host metabolism, immune responses, and inflammatory pathways, central processes in carcinogenesis [27]. Individual responses to the same diet can vary substantially, leading to distinct metabolic and microbial profiles, highlighting the complexity of host–microbiota interactions and the limitations of dietary guidelines [78]. This complexity extends to the characterization of a ‘healthy’ microbiome, which cannot be encapsulated by a single microbial pattern; multiple profiles may support health, with certain taxa exerting protective or pathogenic roles depending on host and environmental contexts [78]. Consequently, there is growing interest in identifying dietary patterns and specific foods that promote beneficial microbial communities and reduce cancer risk. Observational and systematic reviews have consistently linked healthy dietary patterns with a reduced incidence of breast, colorectal, pancreatic, and liver cancers, whereas unhealthy diets correlate with increased cancer risk, like CRC [79]. These epidemiological findings underscore the significant influence that dietary patterns exert on the gut microbiota and its metabolic outputs, which in turn affect cancer risk.

Dietary patterns distinctly affect the gut microbiota and its metabolic outputs. Diet-induced dysbiosis, characterized by reduced microbial diversity and altered metabolite profiles, can disrupt mucosal immune homeostasis and increase intestinal permeability and epithelial stress. This environment favors the production of tumor-promoting compounds and sustains chronic inflammation, creating a vicious cycle that promotes epithelial proliferation, DNA damage, and immune evasion, key steps in tumor initiation and progression [80,81]. Importantly, the impact of diet-induced microbial changes extends beyond the gastrointestinal tract, as microbial alterations have also been implicated in extraintestinal malignancies, such as breast cancer, where dysbiosis affects anticancer metabolite production and estrogen metabolism [76]. Given this broad influence, particular dietary components have garnered attention for their ability to modulate the gut microbiota and confer protective effects against cancer.

Among dietary components, fibers and bioactive compounds in fruits, vegetables, and whole grains play protective roles, partly by modulating the gut microbiota [82]. Fibers, as fermentable substrates reaching the colon intact, enhance microbial diversity and promote production of SCFA such as butyrate [83]. In contrast, low-fiber diets rich in fats and processed foods correlate with reduced microbial diversity and elevated risks of obesity and cancer [84]. In addition to fibers, polyphenols have been shown to selectively stimulate beneficial bacteria while suppressing pathogens, further influencing microbial balance and cancer risk [85]. Polyphenols from tea, chocolate, berries, and other plant-based foods selectively stimulate beneficial bacteria such as *Lactobacillus* and *Bifidobacterium* while suppressing pathogenic taxa including *Clostridium perfringens*, *Clostridioides difficile*, and certain *Bacteroides* species, demonstrating prebiotic and antimicrobial effects [85]. Similarly, intake of flavonoid-rich foods and red wine has been shown to increase *Enterococcus*, *Prevotella*, and *Bacteroides uniformis* without negatively affecting beneficial microbes [86,87]. Building on these microbial shifts, recent research has identified specific species with direct roles in cancer development. For example, *Fusobacterium nucleatum* promotes colorectal tumor progression by enhancing cancer stem cell traits and immune evasion via the IL-17/NF-κB pathway [88,89]. Conversely, probiotics like *Lactobacillus acidophilus* and *Bifidobacterium longum* exhibit antitumor properties by inhibiting tumor growth, reducing inflammation, and restoring epithelial barrier function [90]. Fermented foods rich in beneficial microbes and bioactive peptides, such as yogurt with *Lactobacillus bulgaricus* and *Streptococcus thermophilus*, have been associated with lower colorectal cancer risk, possibly via gut microbiota modulation.

Collectively, these findings emphasize the impact of diet on gut microbiota and its downstream metabolic and inflammatory pathways influencing cancer risk. The therapeutic potential of microbiota-targeted dietary strategies is clear. Future efforts should focus on integrating gut microbial ecology, bioactive food components, and inter-individual variability to develop personalized dietary interventions for cancer prevention and management [21,91].

### 3.1. Mediterranean Diet, Gut Microbiota, and Cancer Prevention

The MD, rich in unprocessed plant-based foods, extra-virgin olive oil, whole grains, legumes, fish, and low in red meat and saturated fats, exerts various effects on gut microbiota composition and functionality. Several meta-analyses and prospective cohort studies have consistently demonstrated that higher adherence to the MD is associated with lower overall and site-specific cancer risk for most types of cancer [92]. Moreover, the protective effect of MD appears to be independent of adiposity, as recent evidence has shown that the inverse association between adherence to the MD and obesity-related cancer risk is not fully explained by differences in BMI or waist-to-hip ratio, suggesting that other mechanisms beyond reduction in abdominal fat might be involved [93]. Various studies have demonstrated that adherence to the MD promotes the enrichment of beneficial microbial taxa such as *Faecalibacterium prausnitzii*, *Roseburia* spp., and *Akkermansia muciniphila*, while reducing the abundance of pro-inflammatory and dysbiosis-associated species, like *Ruminococcus gnavus*, *Collinsella aerofaciens*, and *Ruminococcus torques* [94,95]. These microbial shifts are functionally relevant, enhancing saccharolytic activity and SCFA production, particularly butyrate, which has a potentially relevant for cancer prevention and treatment [95,96]. In addition to reshaping the gut microbial community, the MD exerts direct anticancer effects through its rich content of bioactive compounds, including polyphenols and ω-3 polyunsaturated fatty acids (ω-3 PUFA). These components modulate oxidative stress, promote apoptosis, and regulate tumor suppressor gene expression through epigenetic mechanisms [16,97,98]. Fermented dairy products and polyphenol-rich foods also enhance host immune responses and inhibit pro-tumorigenic inflammation by targeting key signaling pathways, such as NF-κB and STAT3. Furthermore, specific probiotic strains commonly associated with MD, including *Lactobacillus reuteri* and *L. helveticus* R389, have demonstrated efficacy in preclinical breast cancer models, by boosting regulatory T cell function and reducing the production of inflammatory cytokines [99,100]. These immunomodulatory effects correlate with other MD-mediated mechanisms relevant to breast cancer, including modulation of the estrobolome, the gut microbial gene pool involved in estrogen metabolism. Low activity of bacterial β-glucuronidases (GUS), predominantly expressed by *Clostridium*, *E. coli*, *Streptococcus*, and *Bacteroides*, limits estrogen recirculation, thus reducing systemic exposure to carcinogenic estrogens [101,102]. Fiber-rich components of the MD further reduce GUS activity and promote estrogen excretion [101]. As a matter of fact, the PREDIMED trial showed a significant reduction in invasive breast cancer risk among women following an MD enriched with extra-virgin olive oil compared to a low-fat diet [103]. Experimental models confirm these findings, with MD increasing microbial diversity and levels of *Lactobacillus*, *Clostridium*, and *Enterococcus faecalis*, and reducing pro-inflammatory taxa [104]. These microbiota-driven changes have also been demonstrated to be particularly relevant in CRC, where favorable microbial remodeling correlates with significant chemo-preventive outcomes. Longitudinal studies and meta-analyses report an 8–11% risk reduction for CRC with sustained adherence to MD [105,106]. Dietary patterns characterized by higher fiber intake, such as MD and PBD, modulate the gut microbiota, thereby reducing the incidence of *Fusobacterium nucleatum*-enriched CRC in humans [107]. Increased dietary fiber intake also promotes the growth of SCFA-producing genera such as *Clostridium*, *Roseburia*, and *Eubacterium*, which in turn contribute to host protection against advanced colorectal adenoma in a human study [108].

Mechanistically, as previously introduced, SCFA like butyrate inhibit carcinogenesis by promoting regulatory T cell differentiation, enhancing dendritic cell activation, and downregulating oncogenic signaling pathways [105]. Additionally, some studies with MD-based interventions on carcinogenic compound azoxymethane (AOM)-induced models, such as MD-MIX (a formulation enriched with plant polyphenols and Eicosapentaenoic acid), have demonstrated reduced colonic lesions and rebalanced microbial communities, even in pro-inflammatory Western dietary backgrounds [109]. MD also allows the depletion of pro-carcinogenic species such as *Fusobacterium nucleatum*, *E. coli*, and *Clostridium perfringens*, implicated in genotoxicity and immune evasion in CRC [109,110].

In addition to its protective role in CRC carcinogenesis, MD has also been associated with a reduced risk of hepatocellular carcinoma (HCC), a malignancy in which the gut–liver axis plays a central role. Multiple mechanisms underpin this protective effect, including improved insulin sensitivity and reduction in hepatic fat accumulation, particularly relevant in metabolic dysfunction–associated steatotic liver disease (MASLD), a key risk factor for HCC, modulation of bile acid metabolism, suppression of DCA production, and restoration of gut barrier integrity [16,111]. Furthermore, alterations in gut microbiota composition and function have been consistently implicated in HCC development and progression.

Dysbiosis and increased intestinal permeability facilitate the translocation of microbial products such as lipopolysaccharides (LPS), bile acids, SCFA, and endogenous ethanol to the liver, exacerbating hepatic inflammation, fibrosis, and tumor development [112]. MD can counteract these processes by promoting a favorable microbial profile, with increased abundance of *Prevotella* and *Oscillibacter*, and enhanced SCFA production, supporting anti-inflammatory immune responses and hepatocyte protection [113,114]. D-integrated probiotic and prebiotic strategies, such as supplementation with *Lactobacillus* spp., may potentiate these benefits by stimulating microbial fermentation, improving gut barrier function, and modulating cytokine responses [113,114]. Moreover, randomized clinical trials provide evidence supporting the use of probiotics in the prevention and treatment of MASLD and its complications, including MASLD-related hepatocellular carcinoma (MASLD-HCC) [115,116].

Overall, the Mediterranean diet exerts anticancer effects through the combined modulation of gut microbiota and bioactive dietary components. Enrichment of beneficial microbial taxa and increased SCFA production support intestinal barrier integrity, anti-inflammatory responses, and antitumor immunity. These effects, coupled with the actions of polyphenols and ω-3 PUFAs, regulate oxidative stress, apoptosis, and estrogen metabolism, collectively creating an environment less permissive to tumor development, thereby positioning the MD as an effective dietary strategy for cancer prevention.

### 3.2. Plant-Based Diets, Gut Microbiota, and Cancer Prevention

PBD, including vegetarian and vegan patterns, are characterized by high intake of fiber-rich vegetables, legumes, whole grains, fruits, and nuts, with minimal consumption of animal-derived products [79,117]. Although mechanisms partially overlap with those observed for the MD, PBDs also contribute uniquely to cancer prevention through specific bioactive compounds. Phytochemicals such as quercetin, curcumin, baicalin, and resveratrol modulate oncogenic pathways, including the AMPK/PGC-1α axis, which governs mitochondrial function and cellular energy homeostasis [118], potentially relevant to the carcinogenesis process. Furthermore, PBDs consistently promote increased microbial diversity and have been associated with the enrichment of beneficial taxa, already reported in MD, such as *Faecalibacterium prausnitzii*, *Roseburia* spp., *Akkermansia muciniphila*, *Bifidobacterium* spp., *Lactobacillus* spp., *Prevotella* spp., and *Coprococcus* spp., which play key roles in epithelial homeostasis, immune modulation, and SCFA production [79,119,120,121]. These microbial shifts have been linked to reduced risk of colorectal, breast, and hepatocellular carcinoma.

In CRC, the expansion of SCFA producers such as *F. prausnitzii*, *Roseburia* spp., and *Bifidobacterium* spp. is particularly relevant, as butyrate supports colonocyte differentiation, suppresses inflammation via NF-κB inhibition, and promotes apoptosis of malignant cells [122,123]. In contrast, PBDs reduce the abundance of *Fusobacterium nucleatum*, *Escherichia coli pks+*, enterotoxigenic *Bacteroides fragilis*, and *Clostridium symbiosum*, species implicated in genotoxicity, immune evasion, and tumor progression [122,124].

In breast cancer, PBDs as MD influence estrogen metabolism by modulating the estrobolome, as discussed above, notably reducing the abundance of β-glucuronidase-producing bacteria such as *Clostridium* spp., *Bacteroides* spp., and *E. coli*, thereby limiting estrogen recirculation [102]. Concurrently, probiotic species enriched by PBDs, such as *Lactobacillus reuteri*, *L. fermentum*, *L. acidophilus*, *L. casei*, and *Bifidobacterium lactis*, exhibit anti-proliferative activity and promote regulatory T-cell responses in preclinical models of breast cancer [125,126]. An intervention study reported that higher intake of vegetables and dietary fiber was associated with favorable modulation of the gut microbiota, characterized by enhanced isothiocyanate (ITC) excretion, widely investigated for its anticancer properties, and enrichment of *Roseburia*, suggesting a potential contribution to reduced breast cancer risk [127].

Regarding HCC, microbial modulation by PBDs can counteract gut–liver axis dysfunction. Beneficial taxa such as *A. muciniphila*, *Bifidobacterium adolescentis*, and *L. plantarum* improve gut barrier integrity, reduce hepatic lipogenesis, and limit endotoxin-driven hepatic inflammation [123]. Conversely, diets low in fiber and rich in animal fats promote expansion of *Proteobacteria* and bile acid-transforming species (e.g., *Clostridium*), which enhance hepatic inflammation and fibrosis, key drivers of HCC development [110,124]. For example, *Lactobacillus plantarum* AR113 has been shown to attenuate liver injury in aged mice by inhibiting oxidative and endoplasmic reticulum stress. Probiotic and prebiotic strategies within PBDs may thus modulate key inflammatory and metabolic signals implicated in hepatocarcinogenesis [128,129].

In summary, PBDs provide cancer-protective effects not only through microbiota modulation and SCFA production but also via the intake of diverse phytochemicals, fibers, and vegetal proteins. These components uniquely support microbial diversity, limit GUS activity, and suppress pro-carcinogenic species, specifically influencing estrogen metabolism, immune regulation, and intestinal homeostasis, which collectively reduce the risk in particular of colorectal, breast, and liver cancers.

While promising, these findings are largely derived from animal models and small-scale human studies, underscoring the need for more robust clinical trials to confirm the cancer-preventive effects of PBDs and their associated modulation of microbiota.

### 3.3. Ketogenic Diet, Microbiota, and Tumor Immunomodulation

The ketogenic diet (KD), defined by a very low carbohydrate intake (≤30–50 g/day), high fat content, and moderate protein, induces a metabolic state of ketosis leading to the production of ketone bodies as source of energy. In contexts such as fasting, carbohydrate restriction, intense exercise, starvation, or type 1 diabetes, depletion of glycogen stores promotes the hepatic conversion of fatty acids into ketone bodies (acetoacetate, β-hydroxybutyrate, and acetone), which act as alternative energy substrates. This metabolic reprogramming reduces circulating glucose and insulin levels, suppresses insulin/IGF-1 signaling, and enhances lipolysis and mitochondrial fatty acid oxidation as primary energy sources [130]. Originally introduced for the treatment of epilepsy and later adopted for weight loss and metabolic disorders, the KD has recently attracted interest in oncology, as many cancer cells, due to mitochondrial dysfunction and chromosomal instability, exhibit limited capacity to utilize ketones, thus creating a potential therapeutic window [131].

Mechanistically, the metabolic shift induced by KD inhibits oncogenic pathways such as PI3K/Akt/mTOR and Ras/Raf/MEK/ERK, while simultaneously activating AMPK, reinforcing mTOR inhibition and enhancing metabolic stress in tumor cells. This shift may sensitize tumors to conventional therapies and improve treatment tolerance, particularly in malignancies with high glycolytic dependency [132,133]. At the metabolic level, KD also alters amino acid availability, a critical factor in tumors with high anabolic demands [134,135]. In neuroblastoma models, KD increases serum levels of glutamine, glycine, and serine, while reducing essential amino acids and urea cycle intermediates [136]. Conversely, glioma models and patient samples often exhibit elevated glutamate/glutamine levels under KD, highlighting tumor-specific metabolic adaptations [137]. Leucine, an mTOR activator and ketone precursor, adds further complexity: while its restriction can inhibit breast cancer cell growth, its role within KD remains ambiguous and likely context dependent [138,139]. In addition to amino acid metabolism, growing evidence indicates that KD profoundly affects lipid metabolism, which may either suppress or promote tumor progression depending on the cancer context [140]. In pancreatic ductal adenocarcinoma (PDAC), for instance, lipid-driven metabolic plasticity allows cancer cells to adapt to ketone-rich environments. KD can enhance lipid oxidation and phospholipid remodeling, which sustain tumor growth and metastatic spread [141,142,143]. Furthermore, ketone bodies such as β-hydroxybutyrate (βHB) may fuel oxidative phosphorylation in PDAC cells, supporting survival under nutrient-restricted conditions [144]. These findings emphasize the dual nature of KD in cancers like PDAC, where metabolic reprogramming toward lipid and ketone utilization undermines its therapeutic intent. Clinical data on the ketogenic diet (KD) remain limited, with most evidence from case reports. These observations nonetheless suggest potential antineoplastic effects, including reduced tumor glycolysis in selected patients [145]. Notably, in a reported case of breast cancer, a 3-week KD supplemented with olive oil and high-dose vitamin D was associated with favorable modulation of HER2 and progesterone receptor expression, raising the possibility that oleic acid and the KD may have acted synergistically to promote HER2 downregulation [146].

Besides these systemic effects, KD induces significant changes in gut microbiota composition. It typically reduces *Bifidobacterium* and *Actinobacteria* while enriching taxa such as *Akkermansia muciniphila*, *Bacteroidetes*, *Lactobacillus*, and *Roseburia*, which contribute to mucosal barrier integrity and the production of SCFA [147,148]. Among these, *Akkermansia muciniphila* is particularly linked to enhanced mucosal integrity and immune modulation, potentially supporting anti-tumor responses [149]. Similarly, *Roseburia* and *Lactobacillus* species enriched by KD can produce SCFA and reduce intestinal inflammatory cytokine release, further contributing to gut health and systemic immune regulation [148,150]. These microbial shifts have been associated with reduced intestinal Th17 responses and improved anti-tumor immunity, as demonstrated by fecal microbiota transplantation from KD-fed mice [148,151]. However, long-term adherence to KD may lead to a decrease in butyrate levels, potentially impairing colonic barrier function and systemic immune homeostasis [147].

In CRC, *Lactobacillus plantarum* is associated with enhanced barrier protection and reduced proliferation of malignant cells [152,153]. Conversely, the reduction in Bifidobacterium may impair anticancer immunity, as this genus supports T-cell activation and responses to ICIs [154]. Butyrate depletion could further compromise intestinal integrity, increasing susceptibility to inflammation and colon carcinogenesis [147]. Additionally, βHB also exerts direct anti-tumor effects. It is the main circulating ketone body, which shares structural similarities with butyrate and may partially mimic its gut-protective effects, but this functional substitution remains speculative [155,156]. In CRC, it inhibits cancer cell proliferation via activation of HCAR2 signaling and enhances the efficacy of anti-programmed cell death 1 (PD-1) immune checkpoint blockade [144]. These findings underscore how both microbial and metabolic alterations induced by KD can influence tumor behavior, either positively or negatively, depending on the tumor context.

Preclinical and clinical evidence supports KD’s therapeutic promise in various malignancies. In glioma, KD prolongs survival and potentiates the effects of radiotherapy (RT) and temozolomide while preserving healthy brain tissue [157]. In breast cancer, ketone bodies have been shown to inhibit tumor growth, increase survival, and synergize with PI3K inhibitors and rapamycin [158,159]. However, possible pro-metastatic effects via BACH1 upregulation have been observed, though these may be mitigated by specific probiotic strains [160]. In CRC, in addition to βHB-mediated effects, KD has been shown to reduce tumor burden, systemic inflammation, and cancer-associated cachexia [161].

However, KD’s efficacy is not uniform across cancer types. In PDAC, the role of KD is controversial. While it may improve liver function and systemic metabolic parameters, PDAC cells can adapt to utilize ketone bodies as an energy source. In preclinical models, βHB has been shown to promote PDAC progression and liver metastasis, likely via upregulation of ketolytic enzymes such as HMGCL, SCOT, and BDH1 [162,163]. Conversely, suppression of HMGCL reduces tumor growth, suggesting that in PDAC, glucose restriction, not ketone elevation, may be the key therapeutic mechanism [162]. These divergent outcomes highlight the importance of tumor-specific metabolic plasticity and underscore the need for caution when applying KD to metabolically adaptable cancers.

Ultimately, the anticancer potential of the KD appears highly context-dependent, shaped by tumor type, mitochondrial function, metabolic flexibility, host lipid metabolism, and microbiota composition. Its dual impact on the gut microbiome, promoting beneficial taxa such as *Akkermansia* and *Lactobacillus* while depleting *Bifidobacterium* and butyrate, further complicates its clinical translation. In summary, KD may enhance antitumor immunity, sensitize tumors to therapy, and inhibit proliferation in selected contexts, but its benefits can be undermined by butyrate depletion, loss of beneficial microbes, and the metabolic plasticity of cancers such as PDAC. Personalized strategies that integrate host metabolism and microbiota profiling, supported by robust clinical trials, are therefore essential to define when and how KD can be safely and effectively incorporated into oncology care.

### 3.4. Western Diet, Dysbiosis, and Tumor-Promoting Microbiota

The WD, characterized by high intake of saturated fats, red and processed meats, refined carbohydrates, and ultra-processed foods (UPFs), is consistently implicated in the increased risk and progression of several cancers. The direct role of UPFs adds complexity to the cancer scenario. Emulsifiers, sweeteners, and preservatives commonly present in UPFs compromise the intestinal mucus layer, facilitating microbial translocation and immune dysregulation [164,165]. This disruption sustains a pro-tumorigenic microenvironment not only in the gut but also in distant organs such as the breast and pancreas [166,167,168]. Clinically, epidemiological studies consistently link high WD and UPF consumption with increased incidence and worse prognosis in both colon and breast cancers [18,164]. Patient-derived microbiome analyses reveal that WD- and obesity-associated shifts correlate with distinct tumor microenvironment metabolic signatures, including elevated oxidative stress markers and depletion of micronutrients essential for immune regulation, such as niacin [76]. Indeed, this dietary pattern promotes gut microbiota dysbiosis, leading to reduced microbial diversity and a shift toward pro-inflammatory and tumor-promoting taxa such as *Ruminococcus*, *Bacteroides*, *Bilophila*, *Alistipes*, and *Desulfovibrio* [169]. These changes are associated with a decline in SCFA production, particularly butyrate, a key immunomodulatory metabolite [170]. Reduced butyrate levels increase intestinal permeability, facilitating the translocation of bacterial endotoxins such as LPS, which activate NF-κB signaling and promote chronic low-grade inflammation, a hallmark of tumor initiation and progression [171].

In CRC, both clinical and preclinical studies have demonstrated that WD-induced dysbiosis enriches pro-carcinogenic species such as *Fusobacterium nucleatum* and *Peptostreptococcus anaerobius*. These bacteria drive tumorigenesis through immune modulation, production of genotoxic metabolites, and interference with host signaling pathways [18]. Additionally, microbial metabolism of dietary components such as choline, L-carnitine, and phosphatidylcholine (abundant in red meat, dairy products, and eggs) produces trimethylamine (TMA), which is oxidized in the liver to TMAO. This metabolite has been implicated in cardiometabolic and inflammatory diseases [14] and also linked to increased risk of both CRC and PDAC [17,18]. Experimental models confirm that TMAO promotes inflammation and DNA damage in colonic epithelial cells, thus accelerating tumor growth [19]. Mechanistically, TMAO is involved in the activation of the NF-kB signaling pathway and promotes the formation of the NLRP3 inflammasome [15,16], thus contributing to systemic inflammation [14]. An ongoing clinical trial is examining the interplay between the WD, gut microbiota, and colorectal cancer risk (ClinicalTrials.gov: NCT03416777), intending to elucidate the metabolic pathways through which dietary patterns may influence neoplastic susceptibility in otherwise healthy individuals via fecal microbiome [172].

In breast cancer, WD-associated dysbiosis appears to influence systemic inflammation and estrogen metabolism, two key drivers of hormone-dependent tumorigenesis. Indeed, meta-analysis has reported an association between adherence to a WD and an increased risk of breast cancer, particularly among specific populations, such as postmenopausal women [173].

WD has been shown to alter gut microbial composition in animal models, reducing levels of *Lactobacillus* and *Bifidobacterium* species, taxa involved in the deconjugation and recycling of estrogens via the enterohepatic circulation. This microbial shift increases circulating estrogen levels, thereby promoting B cell proliferation. Moreover, WD-induced obesity exacerbates these effects by altering inflammatory profiles in adipose tissue and further disturbing microbial homeostasis [174,175,176]. The impact of WD on lipid metabolism also plays a crucial role, particularly in pancreatic cancer. High-fat WD promotes accumulation of specific lipid species that may support tumor growth and immune evasion [140,177]. Lipid-enriched microenvironments can fuel PDAC progression by supporting mitochondrial β-oxidation and phospholipid remodeling, processes closely linked to cancer cell survival and metastasis [140]. Simultaneously, WD-driven microbial dysbiosis fosters a tumor-promoting milieu in the pancreas: there is frequent expansion of *Bilophila wadsworthia*, associated with taurine-conjugated bile acids, and *Desulfovibrio*, both linked to inflammation and tumor growth [171,178]. WD also selects for *Clostridium scindens* and related *Clostridium* species capable of converting primary to secondary bile acids, such as DCA, which contributes to oxidative stress and activates oncogenic pathways like Ras and NF-κB in the pancreas and colon [179,180,181]. Moreover, WD-associated dysbiosis decreases beneficial species like *Lactobacillus* and *Bifidobacterium*, reducing SCFA production and gut barrier integrity, which may facilitate systemic inflammation and microbial translocation to the pancreas [182]. Some microbial-generated bile acids may activate vitamin D receptor (VDR)-mediated protective pathways in gut-associated cancers, but under WD, the shift favors pro-carcinogenic metabolites [183]. The combination of lipid-driven metabolic reprogramming, secondary bile acid accumulation, and microbiota-mediated inflammation creates a microenvironment that supports PDAC development and progression [184].

Collectively, these findings emphasize the multifaceted impact of the WD on cancer pathogenesis. By driving dysbiosis, WD promotes the expansion of pro-carcinogenic taxa and reduces SCFA producers, leading to impaired barrier function, systemic inflammation, and altered estrogen and lipid metabolism. These changes create a metabolically dysregulated and pro-inflammatory environment that fosters tumor initiation and progression across different cancer types. In contrast to the MD, which enhances microbial diversity, SCFA production, and anti-inflammatory pathways, WD supports carcinogenesis through the convergence of dietary, microbial, and metabolic insults.

**Figure 1:**
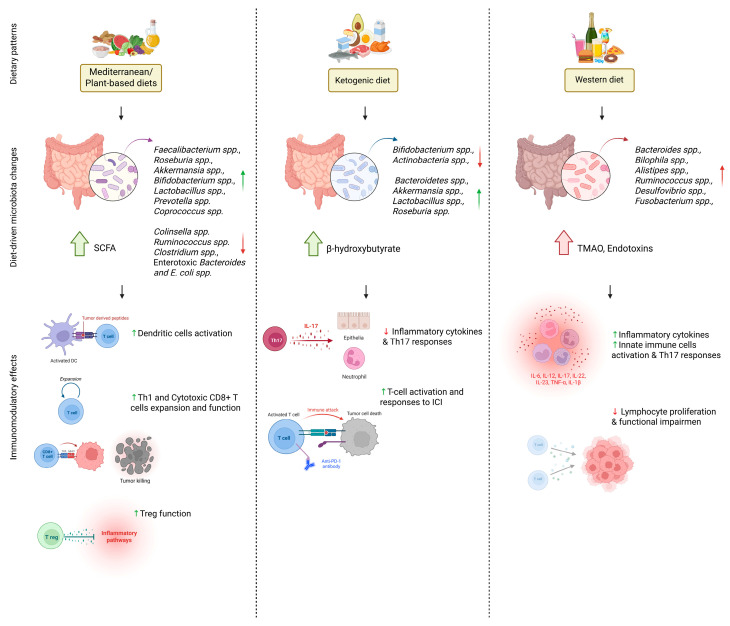
Diet–microbiota interactions and their immunomodulatory effects across different dietary patterns. Abbreviations: TMAO, trimethylamine *N*-oxide; ICI, immune checkpoint inhibitors. The black arrows indicate the correlation between diet, gut microbiota, and immunomodulation. Green arrows indicate an increase; red arrows indicate a decrease.

A comprehensive illustration of how different dietary patterns influence gut microbiota composition and function, and associated immunomodulatory effects, are represented in Figure 1.

## 4. Microbiota and Cancer Therapy

Chemotherapy, RT and targeted therapies, often used in combination with immunotherapy, are cornerstones of cancer treatment. Therefore, understanding the mechanisms by which microbiota can modulate these different therapies by acting on either innate or adaptive immunity is significant.

### 4.1. Microbiota and Chemotherapy

The role of gut microbiota in modulating chemotherapeutic pharmacokinetics, efficacy and toxicity has been deeply investigated [25].

A compelling body of evidence demonstrated that the gut microbiota can modulate chemotherapy efficacy by providing a tumor microenvironment favoring the toxic effect of the drugs on cancer cells and sustaining anticancer adaptive immunity following drug induced immunogenic cell death. In fact, first studies demonstrated that gut microbiota could stimulate antitumor immune responses by modulating CD8^+^ T cells, Th1, and tumor-associated myeloid cells. Conversely, the effects of cancer therapy have been demonstrated to be attenuated in antibiotic-treated or germ-free mouse models [185]

One of the most consistent models of study is CRC, in which the supposed mechanism related to chemotherapy efficacy is the gut microbiota ability to produce ROS, pivotal for cytotoxic damage of DNA. Thus, platinum-based compounds and 5-fluorouracil effectiveness could be related to this effect [186,187].

Likewise, the efficacy of the alkylating agent cyclophosphamide (CTX) could depend on the gut microbiota, with mechanisms related to immunomodulation. CTX induces immunogenic tumor cell death, by pathogenic Th17 and memory Th1 cells [188]. In preclinical models, the translocation of *Enterococcus hirae* to lymph nodes and the accumulation of *Barnesiella intestinihominis* in the colon promoted anticancer immunity induced by CTX treatment [189]. Moreover, CTX reduced bacterial species from the phyla *Firmicutes* and *Spirochaetes* in the small intestine, while increased the abundance of other bacterial taxa, some of which translocated into mesenteric lymph nodes [188].

Besides CRC, the gut microbiota is likely involved in patient response to chemotherapy in extra-intestinal cancer, possibly related to activation or degradation of the therapeutic compounds [190]. Growing evidence suggests that bacteria can metabolize gemcitabine into an inactivated form through a deamination process. Specifically, *Gammaproteobacteria* and *Mycoplasma hyorhinis* could promote gemcitabine resistance in pancreatic and breast cancers, respectively [191,192]. Moreover, breast cancer patients responsive to treatment are characterized by gut microbiota abundance of *Clostridiales*, *Bifidobacteriaceae*, *Turicibacteraceae*, and *Prevotellaceae* [193]. Likewise, a high abundance of *Akkermansia muciniphila* was favorable for enhancing the doxorubicin efficacy in breast cancer [194].

A summary of gut microbiota on chemotherapy efficacy is reported in Table 1.

Furthermore, the role of SCFA butyrate in cancer chemotherapy response has been recently demonstrated. In fact, the level of butyrate was significantly higher in CRC chemotherapy responders [205], with supposed mechanism related to inhibition of glucose metabolism, thus increasing the efficacy of 5-fluorouracil (5-FU) via G-protein coupled receptor 109 a-AKT signaling pathway [206]. The composition of the gut microbiota, and consequentially the derived bacterial metabolites, could be rapidly manipulated by diet [207]. Different dietary patterns (i.e., caloric restriction, intermittent fasting and fasting-mimetic diets (FMD), high-fibers diets, KD, and fermented food) have been studied, and in some cases, have been shown to affect immunity and response to cancer treatment. In mice, caloric restriction increased memory T cell accumulation in the bone marrow, enhancing T cell immunity to tumors [208]. Fermented-food diets increase microbiome diversity and change its composition with an anti-inflammatory effect [207]. High-fibers diets have been shown to slightly affect microbiome diversity expression of immune cells, but a differential effect on endogenous signaling in immune cells has been demonstrated [207]. FMDs have been reported to decrease toxicity and ameliorate chemotherapy response with low effect gut microbiota alterations [209].

### 4.2. Microbiota and Immunotherapy

Gut microbiota alterations may affect both patient response to immunotherapy and the extent of immune-related adverse events. ICIs represent a paradigm-shifting strategy in oncology, designed to potentiate antitumor T cell responses by targeting inhibitory checkpoint pathways or reshaping the tumor microenvironment (TME) [210].

Mounting evidence supports the role of the gut microbiota in the ICIs response in preclinical and clinical studies [12,16]. In recent years, the introduction of ICIs immunotherapy has changed the natural history of solid advanced tumors [211]. ICIs act inhibiting tumor immune escape by targeting PD-1 and its ligand (PD-L1), lymphocyte-activating gene-3 (LAG3), cytotoxic T lymphocyte-associated antigen-4 (CTLA-4), and other targets [212]. Milestone publications in 2015 in animal models first linked the gut microbiota to ICIs response [154,201]. In fact, gut microbiota composition influenced anti-PD-L1 therapy responses [154]. For instance, the abundance of *Bifidobacteria* was correlated to the antitumor efficacy of PD-L1 blockade by enhancing dendritic cell maturation and increasing CD8^+^ T cell priming and accumulation in the tumor microenvironment [154]. From 2018, three human studies published in Science demonstrated that gut microbiota composition and diversity were predictive of the efficacy of ICIs therapy [213,214,215]. Complementary to these findings, fecal microbiota transplantation (FMT) from ICIs responding patients to germ-free or antibiotic-treated mice, was demonstrated to ameliorate tumor control.

**Figure 2:**
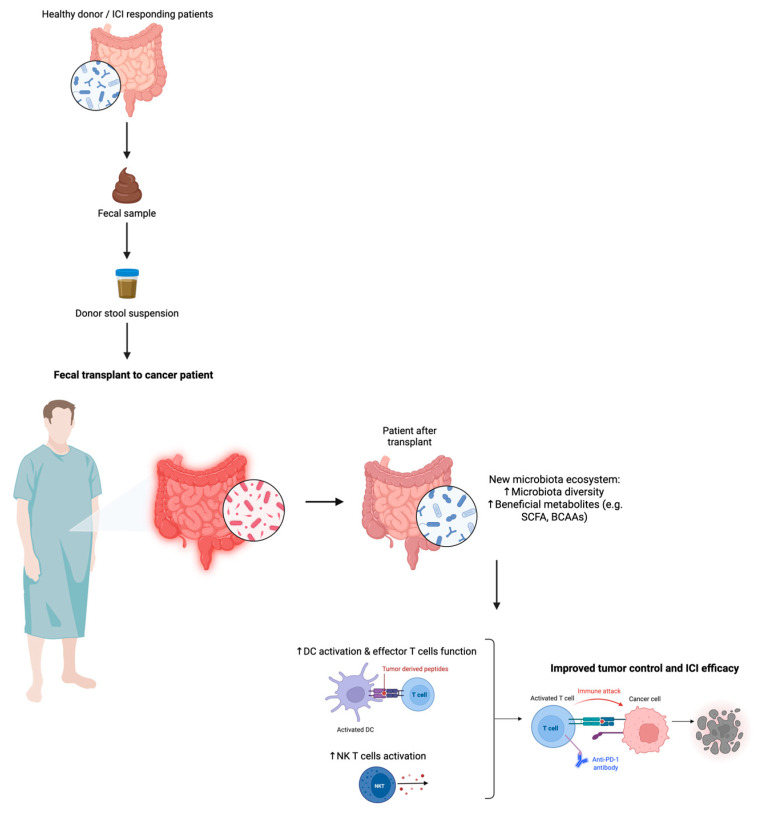
Potential mechanism supporting the role of FMT in cancer immunotherapy. Abbreviations: ICI, immune checkpoint inhibitors; SCFA, short-chain fatty acids; BCAAs, branched-chain amino acids; DC, Dendritic Cells; NK T, Natural Killer T cells; ↑: increase.

In non-small cell lung cancer, renal cell carcinoma, and melanoma, patients with a higher diversity of bacteria were more sensitive to anti-PD-1 therapy [213,214]. In particular, a higher abundance of *Faecalibacterium* and *Ruminococcaceae* in the gut was associated with ICIs response due to increased numbers of CD4^+^ T cells and CD8^+^ T cells [214]. As expected, other studies demonstrated that antibiotics were associated with decreased survival and poor response to ICIs in patients with metastatic solid tumors. Interestingly, two clinical trials in melanoma patients unexpectedly revealed that FMT from ICIs responders combined with anti-PD-1 treatment overcame resistance to PD-1 inhibition [204,215,216]. A representation of potential mechanism underling the role of FMT in cancer immunotherapy is illustrated in Figure 2.

In addition to modulating ICIs immunotherapy, gut microbiota can influence adoptive T cell transfer (ACT) immunotherapy, CpG-oligodeoxynucleotide (CpGODN) immunotherapy, and cell-based immunotherapy.

Evidences suggest that *Clostridiales*, *Lactobacillales*, *Bifidobacteriaceae*, *Akkermansiaceae* are associated with a positive response to anti-PD1 therapy, while *Prevotellaceae*, *Rickenellaceae*, *Bacteroidaceae*, *Proteobacteria* have been linked to poor responses [215,216].

Notably, the SCFA-producing species are associated with a favorable response to ICIs [217]. In fact, SCFA enhance the CD8^+^ cytotoxic activity and CAR T-cells by reducing histone deacetylases and increasing IL-12 responses, leading to the production of interferon-γ (IFNγ) and tumor necrosis factor (TNF), thus concurring in anti-tumor immunity [218,219].

A summary of gut microbiota on immunotherapy efficacy is reported in Table 1.

Considering the effect of diet, in mice treated with anti-PD1, KD improves the antitumor effect through the induction of T cell cancer immunity mediated by 3-hydroxybutyrate [144].

With a different mechanism, fiber sufficient diet was also associated with a better response and survival rate in patients with melanoma treated with anti-PD1 molecules, correlated to changes in the microbiota composition [207,220]. Moreover, a high-fiber diet in mice improve tumor immunity by expanding fiber-fermenting *Ruminococcaceae* spp. with the activation and tumor infiltration by T cells, including inducible T cell co-stimulator (ICOS)-expressing CD8^+^ and CD4^+^ T cells [220].

### 4.3. Microbiota and Radiotherapy

Both bacterial and fungal components of the gut microbiota may contribute to interpatient heterogeneity response to ionizing RT safety and effectiveness [221].

The depletion of the gut microbiota induced by antibiotics is associated with an increase in the gut of the *Saccharomycetes* class of fungi; this shift can reduce RT efficacy by inhibiting antitumor immunity through activation of β-glucan receptor Dectin-1, which induces IL-33 production which, in turn, promotes tumor progression by driving a Th2 immune response [222].

By contrast, treatment with an antifungal drug enhances RT efficiency both in untreated and antibiotic treated mice. The clinical relevance of the immunosuppressive effect of fungi is suggested by the observation that, in patients with breast cancer, expression of Dectin 1 in intra-tumoral cells is associated with a reduction in survival. Moreover, germ free mice are less susceptible to total body irradiation toxicity because the microbiota inhibits the expression of angiopoietin like 4 (ANGPTL4), a lipoprotein lipase inhibitor involved in tissue repair [223]. Moreover, the abundance of bacterial families such as *Lachnospiraceae* and *Enterococcaceae*, which produce tryptophan metabolites and SCFA (particularly propionate), has been associated with less severe intestinal toxicity, with mechanisms related either to mucosal protection [224], and modulation of anticancer immunity via ANGPTL4, IL 18, IL 22 and Treg induction and dendritic cells functions inhibition [225], suggesting a potential regulatory contribution of SCFA in T cell mediated inflammatory effect and intestinal homeostasis.

### 4.4. Fecal Microbiota Transplantation

Fecal microbiota transplantation (FMT) is a proposed treatment of diseases by microbiota manipulation; in particular, it consists of transferring the gut microbiota from healthy donors into the receiving patients to reestablish enteric dysbacteriosis [226]. To date, FMT is approved by FDA for the treatment of recurrent *Clostridium difficile* infection, but its efficacy in chemotherapy modulation is yet to be confirmed. In preclinical research, autologous FMT reduced chemotherapy side effects (MTX- and 5-FU-induced mucositis), by increasing *Lachnospiraceae* and *Roseburia* abundance [227,228].

**Figure 3:**
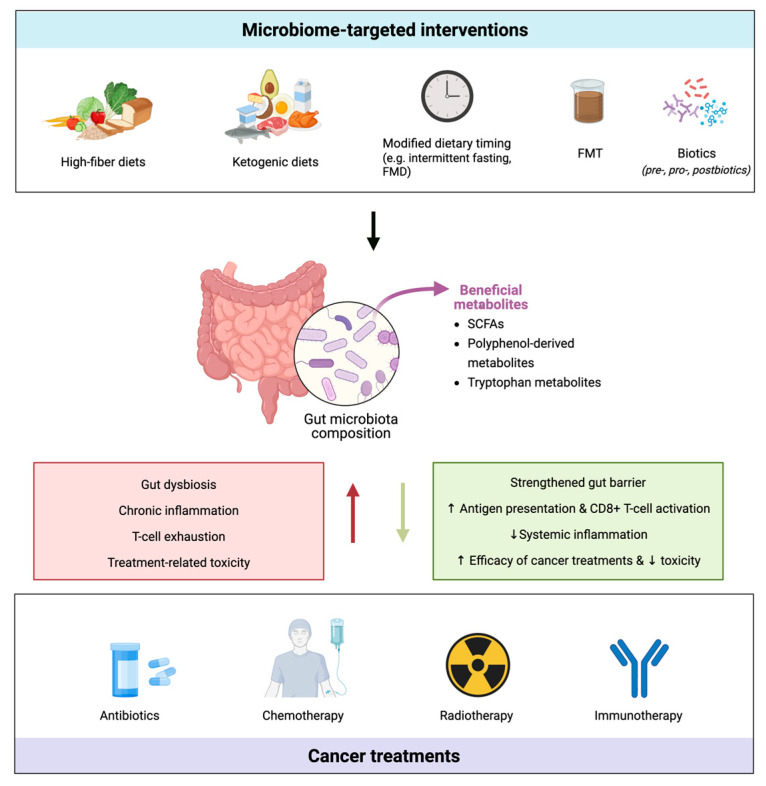
Diet-microbiota-immunity axis in cancer therapy. Abbreviations: FMD, fasting-mimicking diet; FMT, fecal microbiota transplantation. ↑: increase; ↓: decrease.

Another clinical application is related to allogeneic haematopoietic stem cell transplantation (allo- HSCT), in which survival is reduced by life-threatening toxicities, including graft- versus- host disease (GvHD), systemic infections and bacteraemia [229]. Conditioning therapy and antibiotics used prior to allo- HSCT induce mucosal injury, inflammation, immune responses and changes in gut microbiota composition, reducing diversity with single taxon domination [229,230]. A large multicentre study demonstrated that mortality was significantly higher in patients with lower diversity in the gut microbiota before and after the graft [230]. Notably, different antibiotics differentially affect the risk of pathological dominance by *Enterococcus* spp., *Streptococcus* spp. and the *Proteobacteria* phylum [229], while reducing the species of the genus *Blautia* (for antibiotics targeting anaerobic bacteria) [231], and the expansion of *Candida parapsilosis*, associated by elevated transplant-related mortality [232]. On the contrary, higher abundance of *Faecalibacterium*, *Ruminococcus* and *Akkermansia* genera was associated with neutrophil recovery, and *Faecalibacterium* and *Ruminococcus* genera with lymphocytes and monocytes [233]. The administration of *Lactobacillus* spp. was demonstrated to be protective from GvHD in mice, suggesting that the restoration of microbiota eubiosis by fecal microbiota auto-transplantation could improve hematopoietic graft-related toxicity and patient survival [232]. Figure 3 provides an overview of the dynamic regulation mechanism of the diet-microbiota-immunity axis in cancer therapy.

## 5. Diet–Microbiota Interaction in Cancer Survivorship

Beyond its involvement in carcinogenesis, growing evidence further supports the role of diet–microbiota interactions in cancer progression, recurrence, and mortality, as well as in shaping long-term health outcomes in cancer survivors.

### 5.1. Microbiota Modulation by Dietary Components and Patterns: Impact on Cancer Recurrence and Survival

Several studies have demonstrated that both pre- and post-diagnosis diet quality significantly impacts recurrence rates and survival in cancer patients, and systematic reviews and meta-analyses on this relationship have been recently published [234,235,236,237,238,239,240,241]. Overall, adherence to high-quality diets, including MD-style diets characterized by greater intake of fruits, vegetables, whole grains, lean proteins and polyunsaturated fat, as opposed to Western-style diets, has been associated with reduced recurrence risks and improved overall survival across various cohorts of cancer survivors [234,237,242,243,244,245,246,247,248], likely through modulation of the gut microbiome.

Among dietary components of the American Cancer Society (ACS) Nutrition and Physical Activity Guidelines for Cancer Survivors and The Third Expert Report on Diet, Nutrition, Physical Activity and Cancer from the World Cancer Research Fund/American Institute for Cancer Research (WCRF/AICR) network-recommending a healthy diet rich in fruits, vegetables, and whole grains [247,249], a greater quantity and variety of fruit and vegetable consumption was associated with significant variations in the gut microbiome of CRC survivors, including increased microbiota diversity, decreased *Bacteroidota philum*, and lower abundance of *Fusobacterium nucleatum* (implicated in oral, pancreatic, and CRC carcinogenesis), as well as enrichment of several microbial metabolic pathways, such as for amino acids and SCFA biosynthesis, and plant-associated sugar degradation [250].

High-fiber diets can promote fiber-fermenting bacteria producing SCFA with recognized tumor-suppressive effects [251], and increased fiber intake after cancer diagnosis, especially from cereals, has been associated with better survival in nonmetastatic CRC patients [252]. Consistently, fecal microbiome diversity and abundance of certain SCFA-producing bacteria (order *Clostridiales*, *Coprococcus* and *Roseburia*) were strongly associated with improved disease-free survival in stage I–III CRC patients [253]. Similarly, a high dietary fiber intake (>20 g/day), mainly correlated with greater consumption of fruits, vegetables, and whole grain, was associated with improved progression-free survival in melanoma patients. Specifically, a 5 g increase in daily fiber corresponded with a 30% reduction in the risk of cancer progression or death [220]. Microbiome analysis revealed an enrichment in bacterial taxa such as the *Ruminococcaceae* family and the *Faecalibacterium* genus in patients with sufficient fiber intake and no probiotic use undergoing ICIs treatment. Furthermore, in preclinical melanoma models, mice treated with ICIs and receiving a high-fiber diet showed delayed tumor growth, along with increased levels of the SCFA propionate in stool, and higher frequency of activated T cells in the tumors, suggesting that gut microbial-mediated fermentation of fiber and formation of SCFA may contribute to the effects of fiber on antitumor immunity, thereby improving cancer outcomes [209]. Dietary patterns rich in fiber can also increase the abundance of beneficial bacteria such as *Prevotella*, which has been very recently associated with better disease-free survival in breast cancer patients [254]. Moreover, higher *Prevotella* abundance correlated with lower levels of the pro-inflammatory cytokine IL-1β, and patients with low *Prevotella* abundance and high IL-1β levels showed a higher risk of breast cancer recurrence, further supporting the close relationship among diet-gut microbiota-inflammation and its potential prognostic value in cancer patients [254]. Intriguingly, the enrichment in *Prevotella* has been associated with better outcomes in people with obesity following an MD [255]. Since the link between obesity and cancer recurrence in women with breast cancer is well known, the role of bacteria such as *Prevotella* could be one of implicated mechanisms and a target for prevention [256].

Dietary interventions with rice and beans have also been investigated for their potential ability to promote a gut microbial shape relevant for cancer outcomes, especially in patients with CRC. In two 4-week randomized-controlled trials in CRC survivors with overweight or obesity at high risk for recurrence, increased consumption of navy beans (35 g bean powder/day) induced significant changes in plasma, urine, and stool metabolomes, with enrichment in several compounds linked to both host and gut microbial metabolism and implicated in cancer control and prevention [257,258]. In another randomized clinical trial in 29 CRC survivors, supplementation with either rice bran or navy bean powder for 4 weeks led to increased gut microbiota richness, although only rice bran intake resulted in significant changes in gut microbiota diversity and composition [259]. In particular, rice bran supplementation decreased the *Firmicutes:Bacteroidetes* ratio after 14 days due to increased abundance of *Bacteroidetes* and reduced abundance of *Firmicutes*, which may provide protection against CRC [259]. Enrichment of SCFA-producing bacteria, such as of the *Lachnospiraceae* family, has also been reported after rice bran supplementation in both CRC murine models and human CRC survivors [259,260]; however, this was not consistently associated with increased fecal SCFA levels across human studies, possibly due to direct SCFA uptake by colonocytes [119,259,260]. Furthermore, rice brain supplementation demonstrated integrated fecal microbiome and metabolome changes in mice with colitis-associated CRC and adult CRC survivors, with notable shifts in fatty acids, phenolics, amino acids, and vitamins that may contribute to rice’s protective properties against CRC [260,261]. For instance, higher levels of enterolactone, a microbial end-product derived from the metabolism of lignans mainly present in flax and found increased in stool after both rice and navy beans consumption [258,260] have been associated with lower mortality risk and improved survival outcomes in various types of cancer, including colon, lung, prostate, and breast cancers [262] and may thus serve as a promising microbiome-derived biomarker of prognosis.

Specific food components have also shown beneficial associations with cancer outcomes, potentially by modulating systemic inflammation and favorably shaping microbiota composition. For instance, high marine ω-3 PUFA consumption post-CRC diagnosis was associated with reduced CRC-specific mortality [263] and circulating levels of ω-3 PUFA correlated with favorable gut microbiota compositions characterized by increased abundance of Bifidobacterium in breast cancer survivors [264].

Exercise has been demonstrated to independently influence the gut microbiota [265,266] and higher postdiagnosis physical activity has been linked to lower cancer specific- and overall mortality, and lower risk of recurrence in several types of cancer [247]. Moreover, regular physical activity during and after cancer treatment has been shown to positively impact multiple health-related outcomes in cancer patients, including anxiety, depression, fatigue and quality of life [248], thus reinforcing the potential clinical benefits of targeting the microbiota through comprehensive lifestyle interventions in this population. In line with this, a 12-week home-based lifestyle intervention (based on an MD combined with an exercise training program) in breast cancer survivors induced favorable changes in gut microbiota composition able to promote anti-inflammatory profiles, as well as beneficial metabolic effects relevant to health maintenance and prevention of recurrence risk [267]. These included a significant decrease in *Proteobacteria*, an increase in *Lactobacillales* and a decrease in *Sutterella* at the phylum, order and genus level, respectively; less robust non-significant increases were also noted for the *Firmicutes/Bacteroidetes* ratio and *Actinobacteria* (which include *Bifidobacteria*) [267].

Besides conventional healthy/MD-style dietary patterns, other specific dietary regimes, such as low-carbohydrate (LCD), KD, and FMD, have garnered increasing attention in recent years as potential strategies to modulate cancer outcomes. However, the complex interplay between these dietary interventions and the gut microbiome/metabolome in cancer patients remains largely unexplored. In a randomized controlled trial in patients with prostate cancers, a 6-month low-carbohydrate intervention (carbohydrate intake: ≤20 g/day) led to significant weight loss and improvements in cardiometabolic parameters, and was also associated with longer PSA doubling time, suggesting a possible impact on disease progression [268]. Similarly, in an open-label non-randomized intervention trial investigating the effects of 3 different diets (healthy standard diet, LCD, and KD) in breast cancer survivors, adherence to all dietary patterns significantly improved body composition, metabolic markers, physical performance and self-reported quality of life, though the most relevant improvements were observed within the LCD and KD groups. Nonetheless, cancer–specific outcomes were not examined in this study [269]. Preclinical studies and few clinical trials have demonstrated that intermitting fasting and FMD may enhance the efficacy of standard anticancer treatments, mainly by modulating systemic metabolism, growth factors concentrations (i.e., insulin and IGF-1 levels), and antitumor immunity, with potential implications also for cancer risk and recurrence [209,270]. However, all these studies primarily focused on short-term outcomes and/or immediate treatment responses. Therefore, further research is needed to clarify the long-term effects of such more radical dietary strategies on cancer recurrence in survivors as well as to validate their long-term safety and tolerability in this population. Furthermore, recent studies switch on the light on the metabolic reprogramming of cancer cells to sustain their survival in different nutrient milieu, suggesting that tailored dietary programs with or without molecules able to control diet specific by products should be taken into account, in particular in survivorship [271].

### 5.2. Microbiota Modulation by Diet and Lifestyle: Impact on Comorbidities and Quality of Life in Cancer Survivors

Apart from recurrence and survival, emerging evidence highlights a significant role of diet–microbiota interaction in influencing a wide range of comorbidities experienced by cancer survivors, such as cardiometabolic disorders, gastrointestinal symptoms, chronic pain, fatigue, and cognitive impairment, all of which can negatively impact psychological health and overall quality of life.

Persistent intestinal dysbiosis after cancer treatment (mainly chemotherapy and RT, often associated with broad-spectrum antibiotics as prophylactic or therapeutic agents against infections) has been implicated in the development of cardiometabolic disorders in childhood cancer survivors, possibly by fueling peripheral chronic inflammation and promoting visceral fat accumulation, suggesting that early modifications of the microbiota may impact on long-term cardiometabolic risk in this population [22]. In this view, dietary interventions may represent an attractive strategy to restore microbial imbalance and improve patients’ outcomes. Indeed, a growing body of evidence has shown that maintaining or adopting healthier dietary patterns, including traditional MD, the Dietary Approach to Stop Hypertension (DASH) and PBD, can substantially reduce the risk of cardiometabolic conditions (i.e., hypertension, diabetes, and dyslipidemia) and cardiovascular mortality rates among cancer survivors [272,273,274], as already demonstrated in the general population [275,276,277]. Furthermore, it has been demonstrated that the protective associations between MD-style dietary patterns and cardiometabolic health is modulated by specific microbial profiles [96]. However, very few studies have specifically addressed the interplay between nutrition, gut microbiota and cardiometabolic outcomes in the cancer survivor population. In a study comprising 34 overweight breast cancer survivors, an MD combined with probiotics increased bacterial diversity and positively influenced gut microbiota composition, leading to significant improvements in anthropometric, metabolic, and inflammatory parameters, with potential implication for overall cardiometabolic health [278]. Conversely, preclinical evidence derived from studies in mice suggest that poor dietary patterns, such as high-fat diets, may exacerbate the detrimental effects of irradiation on gut dysbiosis and adipose tissue inflammation and metabolic dysfunction, thus acting as a major predisposing factor for the development of metabolic complications in cancer survivors exposed to RT [23].

Associations linking the gut microbiota with gastrointestinal health, cognitive functions and psychological well-being in survivors are also intriguing. In the Chemo-Gut Study, higher self-rated diet healthiness among 334 survivors of different cancers who had completed anticancer therapies was associated with lower GI symptoms and specific microbial signatures, including a higher abundance of *Lachnospiraceae* and a lower abundance of *Bacteroides*, which have been implicated in inflammatory bowel disease [279]. An overall benefit of probiotic supplementation (mainly *Lactobacillus* and *Bifidobacterirum* species) on the incidence of treatment-induced gastrointestinal toxicity and diarrhea has also been reported, thus reinforcing the potential of microbiota-targeted interventions to mitigate survivors’ gastrointestinal symptoms [280,281]. Cancer-related fatigue (CRF) is another common debilitating symptom in cancer survivors and emerging evidence suggests that poor dietary quality, characterized by low intake of protein, fiber, vitamins, and minerals, may contribute to CRF via gut microbiota-mediated mechanisms [24]. Indeed, lower microbial diversity, unhealthier gut microbiota composition, and altered microbial metabolic pathway have been consistently observed across different cohorts of CRF patients, ranging from solid tumor to lymphoma survivors [24]. Specifically, patients with CRF often display a decreased abundance reduced abundance of SCFA-producing taxa (e.g., Ruminococcaceae, *Faecalibacterium*, *Lachnospiraceae*, *Eubacterium*) along with higher abundance of pro-inflammatory taxa [24,282] and alterations in tryptophan metabolism (e.g., lower tryptophan levels and increased kynurenine/tryptophan ratio) [24,283,284], suggesting a mechanistic link between inflammation and CRF via the gut–brain axis. Similarly, diets rich in fiber, polyphenols and healthy fats (including ω-3 PUFA) as well as probiotics promoting a gut microbial profile associated with increased production of SCFA and reduced systemic inflammation may help mitigate cancer-related cognitive impairment, psychoneurological symptoms, and chronic pain, though direct interventional evidence is still limited in this context [281,285,286,287,288,289,290]. Interestingly, a pilot study investigating the effect of exercise in breast cancer survivors revealed significant associations between gut microbiota β-diversity and longitudinal changes in fatigue, anxiety, and cardio-respiratory fitness, outlining the potential contribution of exercise, along with nutritional strategies, in influencing psychosocial outcomes, likely through modulation of the gut–brain axis [290].

**Figure 4:**
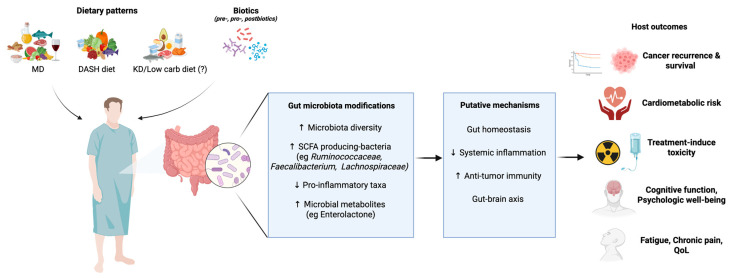
The effects of gut microbiome modification by different dietary patterns and/or biotics in cancer survivors. Created in https://BioRender.com. Abbreviations: MD, Mediterranean Diet; KD, Ketogenic Diet; SCFA, short-chain fatty acids. ↑: increase; ↓ : decrease.

The effects of gut microbiome modification by different dietary patterns and/or biotics in cancer survivors are summarized in Figure 4.

## 6. Limitations and Future Perspectives

Evidence from previous sections highlights as the diet-microbiota axis plays an active role in any step of cancer history, from prevention or pro-tumorigenic effects to therapy response and also survivorship. However, although current data suggest a pivotal role of the gut microbiota as a mediator between diet and survivorship outcomes, most findings mainly derive from a limited number of short-term investigations conducted in small cohorts of cancer patients, generally graded as low to very low in quality. Moreover, available data from these studies often overlap in their definition of “survivors”, dietary data collection, duration of dietary exposures, and outcomes assessed. Existing research, also predominantly focused on common tumors, such as breast, colorectal, and prostate cancers, thus limiting the generalizability of findings to other cancer types. Furthermore, several studies concentrate on specific foods or isolated food components rather than overall dietary patterns, which reduces the applicability for implementing practical dietary recommendations tailored to patients’ needs. Finally, although many cancer survivors report using dietary supplements in the hope of improving quality of life, alleviating treatment-related symptoms and/or disease outcomes, evidence of benefits remains limited, and places emphasis on whole-diet approaches rather than isolated nutrients [291]. Providing a compelling rationale and novelty for developing and integrating microbiota-informed dietary strategies into personalized cancer care is crucial to enhancing the therapeutic response of existing cancer treatments.

As clearly outlined in the previous chapters, healthy diets (MD or PBD) are able to modulate microbiota signatures interacting with host immunity and metabolism, and cancer microenvironment. The emerging data on microbiota composition after them reinforce recommendations from the World Cancer Research Fund [292,293]. Providing patients with information on how diet interacts with microbiota could be a useful strategy to implement adherence to healthy lifestyle habits, since the attrition rate is very high even when tailored programs for weight management and dietary improvements are provided to cancer patients [294].

On the other hand, patients’ microbiota signatures at diagnosis or during their follow-up could be used in the future to give specific advice related to dietary regimens in relation to the type of cancer and proposed treatments, with the aim of increasing their efficacy. However, several hurdles are currently on the street, since microbiota is naturally dynamic and many factors apart from diet could modulate its composition and richness, including age, geographical area, pollution, and drugs. Moreover, signatures could be modulated by prebiotics, conventional and non-conventional probiotics, their mixture, or postbiotics to favor microbial diversity or a certain microbiota consortium with leading functions in relation to both the host and the cancer metabolism [295]. In this landscape, it is important to remember that frequently exciting results in experiments performed in mouse models are followed by non-significant results in clinical trials. Several reasons could contribute alone or together to these negative results, including diminished microbial stability in the human gut, transit time in the gut, and colonization resistance in patients with certain tumor or specific microbial signature before the probiotic administration [295,296,297]. Indeed, researchers should focus on the identification of tailored microbiota-based therapies personalized to the characteristics and needs of each patient. To do this, culture on chips, organoids, gut–ex vivo systems, and inoculation in germ-free animals could be used as preliminary steps before implementing a microbiota-based approach.

Furthermore, live probiotics could be associated with a certain risk of sepsis or transfer of resistance to antibiotics, particularly in immunocompromised patients or those under treatments that could impact on the immune system [298]. The balance between the risk and the benefits could be a further element that drives the choice among specific strains, prebiotics, engineered bacteria, or FMT coupled with specific dietary advice.

Solid tumors are characterized by hypoxic regions and necrotic areas that can be suitable niches for anaerobic bacteria, as demonstrated for *Bifidobacterium*, *Clostridium*, *Salmonella*, and *Escherichia coli*, among others [297]. However, determining whether they are true drivers or merely opportunistic agents adapted to the tumor microenvironment is challenging without longitudinal data from pre-cancer lesions to cancer lesions or experiments in gnotobiotic mice, which are quite resistant to tumor development [295].

Nonetheless, the tumor’s favorable habitat for anaerobic bacteria is a double-edged sword for the tumor itself, since engineered live biotherapeutics could colonize it and secrete molecules, deliver antigens modulating the local immune system, eliminate pro-inflammatory taxa, and detoxify the local microenvironment [295,297,299]. In this view, *E. coli* Nissle 1917 (EcN) has been engineered in several ways for probiotic outcomes in specific diseases, including cancer. Some examples include EcN modification to serve as an immunotherapy by suppressing tumor growth through ammonia production or modulating T-cell metabolism and anti-tumor activity through metabolization of ammonia to L-arginine [300,301].

*Lactobacilli*, known to be safe, have also begun to be engineered. In particular, *Lactococcus lactis* NZ9000 has been modified for various therapeutic applications, including being used as a vaccine delivery system for antigenic display in cancer immunotherapy [302]. Other companies developed different strategies. For instance, a consortium of 11 human commensal bacteria strains, named VE800, is under investigation in combination with nivolumab (a currently used ICI) with the aim of inducing CD8^+^ T cells and contributing to tumor suppression in advanced metastatic cancers (ClinicalTrials.gov identifier: NCT04208958) [303]. These strategies could be combined in the future with metabolic end products of bacteria or their surface metabolites having immunomodulatory effects, potentially useful during immunotherapy with ICI [299,304].

FMT is a further exciting treatment option to be coupled with standard anticancer therapies as well as immunotherapy. Intriguingly, FMT from patients treated with ICI in recipient mice with induced cancer resulted in response or no response in line with those of donors in almost all cases [299]. Proof of concept studies in melanoma patients have shown that FMT from patients with a complete response to ICI could overcome or blunt the resistance to the treatment in host patients who do not respond to ICI immunotherapy [304]. Intriguingly, the response to ICI in recipient mice improved after FMT from obese patients who had undergone bariatric surgery, resulting in reduced tumor burden and doubled immunotherapy effectiveness. These effects seem partly mediated by several metabolites produced by the new microbiota ecosystem, in particular BCAAs, able to increase and activate natural killer T cells [305]. However, FMT is at least in early stages of clinical development in patients with cancer. The risk of transmission of multidrug-resistant species and secondary adverse effects on other health outcomes (susceptibility to infection, immunity reactions, metabolic alterations) should be taken into account and could be partially dependent on an individual’s baseline microbiota signature. Liquid biopsies and machine learning algorithms could revolutionize cancer detection and management, including the application of FMT or the previously described strategies.

## 7. Conclusions

Microbiota is a key player in any stage of cancer history, from prevention to treatment and survivorship. We could imagine that microbiota will enter in the near future in the staging of patients or in the choice of treatments. Microbiota and its metabolites are emerging as features to be considered in this landscape and their modulation through multilevel strategies (tailored diets, antibiotics, standard or engineered probiotics, prebiotics, postbiotics or FMTs) are challenges for research and clinical practice.

In upcoming years, biotherapeutic products for the treatment of human diseases, including cancers, and bacterial therapy should enter the pipeline of biotechnology companies and research institutions. Guidelines for microbiome sampling, sequencing, and data analysis have been recently published to enhance the comparability of findings across studies and the identification of therapeutic strategies [306]. More strengths are needed to integrate machine learning and other Artificial Intelligence techniques to efficiently analyze and integrate microbiome datasets, including their metabolic products. These efforts may help to identify microbial signatures that can predict the risk of cancer and the best biotherapeutic treatment to be combined with standard cancer therapies.

## Figures and Tables

**Table 1:** Effects of the gut microbiota on chemotherapy/immunotherapy efficacy.

Therapeutic Molecule	Mechanism	Bacteria	Gut Microbiota Effect on Anticancer Therapy	References
Chemotherapies				
Cyclophosphamide	Alkylating agent	*Lactobacillus johnsonii*, *L. murinus*, *Enterococcus hirae*, *Barnesiella intestinihominis*	↑ therapeutic response via gut translocation **→** secondary lymphoid organs; ↑ Th1 and Th17 induction.	[188,189]
Cisplatin/Oxaliplatin	Platinum based agents	*Alcaligenes*, *Lactobacillus*, *Alistipes*	↑ therapeutic response via TLR4 activation **→** ↑ ROS production mediated by myeloid cells	[186]
Gemcitabine	Nucleoside analogs	*Mycoplasma hyorhinis*	↓ therapeutic response via enzymatic drug degradation **→** ↓ cytostatic activity	[192,195]
Paclitaxel	Taxane	*Roseburia*, *Eubacterium*, *Erysipelotrichaceae*	↓ bacteria diversity and ecological network function; ↓ butyric-producing bacteria	[192,196]
CPT-11 (Irinotecan)	DNA topoisomerase I inhibitor	*Escherichia Coli*	↑ GI toxicity via SN-38-G conversion into SN-38 induced Bacterial β-glucuronidase; ↓ treatment tolerability	[197,198]
Carmustine, Etoposide, Aracytine and Melphalan combination	Conditioning chemotherapy (HSCT)	*Firmicutes*, *Enterococcaceae Actinobacteria*, *Proteobacteria*	↓ diversity and ecological network function; ↑ drug toxicity; ↑ inflammation (colitis)	[199]
Methotrexate	Antimetabolite	*Clostridium*, *Eubacterium*, *Bifidobacteria*, *Bacteorides (Anaerobes).* *Lactobacilli*, *Streptococci*, *Enterobacteriaceae (Aerobes)*	↓ microbial diversity ↑ pathogenic taxa **→** mucositis, inflammation, and GI toxicity.	[200]
Immunotherapies				
CTLA-4 blockade (Ipilimumab)	Anti- CTLA-4 antibody	*Bacteroidetes*	↑ treatment response via CD4^+^ T cell activation effector T cells. ↓ colitis via polyamine/vitamin B modulation	[201,202]
Anti-PD-L1	Checkpoint blockade	*Bifidobacterium* spp.	↑ treatment response via tumor-specific T-cell induction; ↑ CD8^+^ infiltration in tumour microenvironment	[154,201,203]
Anti-PD1	Checkpoint blockade	*Lachnospiraceae*, *Ruminococcaceae*, *Bifidobacteriaceae*, *Coriobacteriaceae*	↑ treatment response via CD8^+^ T cell and innate effectors activation; ↓ suppressive myeloid cells	[204]

Legend. The arrow indicates the increase or decrease of the microorganisms ↑: increase ; ↓: decrease.

## Data Availability

No new data were created or analyzed in this study.
